# Dear Doctor

**Published:** 1894-04

**Authors:** E. P. Cunningham

**Affiliations:** Goshen, Ind.


### CORRESPONDENCE.

GOSHEN, IND., March 28, 1894.

*Dear Doctor*:—I wish to inform you of a death produced by the injection of cocaine for the extraction of teeth.

It occurred in the office of a dentist of this place to-day. He is an excellent dentist and a man of thirty years experience. The solution used was taken from a formula recommended by, and published in the January number of the *Dental Practitioner and Advertiser*. I was summoned to the office with physicians soon after the toxic effects were noticed to be serious. The Dr. had injected about 10 min. of the solution, and had extracted two badly-decayed molars, when the patient complained of some weakening effects. The usual toxic effects produced when too large a dose has been administered, as you understand much better than I do. All known restoratives were administered, but with no effect.

The Dr., I can assure you, is a careful man and a student of your friend, I believe, Dr. Brown, of Fort Wayne. I write you the facts of the case thinking that it may interest you, and to prevent any false publication. The patient was the Cashier of the First National Bank, and a man who has long been a sufferer from rheumatism, and is about fifty years of age. I desire that no responsibility be thrown upon the dentist, as he was not to blame. Some valvular heart trouble has also been diagnosed by his physician, from which he has also suffered.

Yours respectfully, E. P. CUNNINGHAM.

